# Epidemic Trends and Associated Factors of the Double Burden of Malnutrition Among Children and Adolescents in Xizang: Based on Five Cross-Sectional Surveys from 2019 to 2024

**DOI:** 10.3390/nu18203340

**Published:** 2026-10-12

**Authors:** Dengcheng Zhang, Jianuo Jiang, Liming Tan, Zhuoma Pubu, Sujuan Li, Qiang Zhang, Li Chen, Jinghong Liang, Xinli Song, Yi Zhang, Ruolin Wang, Ziqi Dong, Yang Qin, Zhenting Lu, Jun Ma, Yanhui Dong

**Affiliations:** 1 Xizang Center for Disease Control and Prevention, Lhasa 850000, China; zhangdc815@bjmu.edu.cn (D.Z.); 954510884@qq.com (L.T.); 1245861821@qq.com (Z.P.); 394562337@qq.com (S.L.); 809691012@qq.com (Q.Z.); 2 Institute of Child and Adolescent Health & School of Public Health, Peking University; National Health Commission Key Laboratory of Reproductive Health, Beijing 100191, China; 1810306143@pku.edu.cn (J.J.); clcl@bjmu.edu.cn (L.C.); liangjh78@mail2.sysu.edu.cn (J.L.); xinlisong1996@163.com (X.S.); zy1999pku@pku.edu.cn (Y.Z.); ruolinwang0529@163.com (R.W.); 1910306215@pku.edu.cn (Z.D.); thomasqin@bjmu.edu.cn (Y.Q.); 2010306228@stu.pku.edu.cn (Z.L.); majunt@bjmu.edu.cn (J.M.)

**Keywords:** double burden of malnutrition, children and adolescents, Xizang, epidemic trends, associated factors

## Abstract

**Introduction:** The population-level double burden of malnutrition (DBM), characterized by the coexistence of undernutrition and overnutrition, presents a complex public health challenge. Xizang (Tibet), with its extreme high-altitude geography, rapid economic growth, and targeted school health policies, offers a unique context for nutritional transition, yet large-scale, repeated studies on the region’s DBM trends and multi-level associated factors remain scarce. **Methods:** Data were obtained from five large-scale cross-sectional surveys conducted in Xizang between 2019 and 2024 (except for 2022, when surveillance was not conducted), covering 301,242 participants aged 6–17 years. Nutritional status was defined using WHO growth standards for stunting, thinness, overweight, and obesity. Children and adolescents with stunting or thinness were classified as having undernutrition, while those with overweight or obesity were classified as having overnutrition. Prevalence was standardized across survey cycles—overall and within demographic, geographic, and socioeconomic strata—using multivariable logistic regression models to control for potential compositional variations. Multivariable mixed-effects models (MMEM) were used to analyze associations between potential correlates and the odds of undernutrition and overnutrition. **Results:** From 2019 to 2024, the standardized prevalence of undernutrition markedly decreased from 11.30% to 6.09%, driven primarily by stunting (9.42% to 4.12%). Overnutrition standardized prevalence peaked at 12.10% in 2020 (up from 9.42% in 2019), dipped to 11.24% in 2021, rebounded briefly to 11.44% in 2023, and fell to 10.00% in 2024. The standardized prevalence of undernutrition steadily increased with age and was consistently higher among boys, middle school students, and populations in rural, less developed, and high-altitude regions. Conversely, overnutrition decreased with age and predominated among girls, primary school students, and residents of urban, low-altitude, or moderately developed regions. Odds of undernutrition and overnutrition were significantly associated with a multi-level array of factors, including individual behaviors, school health management, socioeconomic status, altitude, and environmental variables. **Conclusions:** Xizang exhibits a unique “high-altitude DBM” pattern. The findings emphasize that public health strategies must integrate individual behavioral interventions with school-based health management and a nuanced understanding of environmental influences to effectively address the diverse nutritional needs of high-altitude populations.

## 1. Introduction

The double burden of malnutrition (DBM) is characterized by the coexistence of undernutrition alongside overweight and obesity, or diet-related noncommunicable diseases, within individuals, households, and populations, and across the life course [1]. In the present study, DBM is operationally defined at the population level, characterized by the co-occurrence of undernutrition (stunting and thinness) and overnutrition (overweight and obesity) within the surveyed pediatric population. In this context, undernutrition serves as an overarching category encompassing both stunting (linear growth deficit, height-for-age z-score < −2) and thinness (body mass deficit, BMI-for-age z-score < −2), whereas overnutrition broadly covers overweight (1 < BMI-for-age z-score ≤ 2) and obesity (BMI-for-age z-score > 2).

From 1990 to 2021, the global prevalence of overweight and obesity doubled among 5–14 year-olds (8.8% to 18.1%) and 15–24 year-olds (9.9% to 20.3%), totaling 493 million affected young people in 2021 [2]. With regard to undernutrition, although its prevalence has declined substantially since the early 1990s, it remains high in South Asia and parts of Africa [3]. Chinese children and adolescents are simultaneously facing the dual nutritional stress of “persistent undernutrition and surging overweight/obesity” [2,4]. Results from the Chinese National Survey on Students’ Constitution and Health (CNSSCH) show that among Chinese children and adolescents aged 7–18 years, the overall detection rate of overweight and obesity increased steadily from 1.2% in 1985 to 23.4% in 2019 [5]. Meanwhile, the detection rate of undernutrition dropped from 12.7% in 2010 to 10.1% in 2014 and 8.5% in 2019 but slightly increased in the South and Northwest regions [6].

Located in the core of the Qinghai–Tibet Plateau in western China, the Xizang Autonomous Region possesses a unique natural environment and a relatively nascent socioeconomic status, both of which may profoundly influence the nutritional landscape of its residents [7,8]. A prior study evaluating the Nutrition Improvement Program for Rural Compulsory Education Students across 45 counties in Xizang from 2015 to 2017 revealed that the prevalence of thinness and stunting among students aged 6–16 remained higher than the national average [9]. Concurrently, the rates of overweight and obesity rose annually, signaling the emergence of the DBM. Moreover, a 2018 survey conducted in Xigaze, Xizang, showed that the prevalence of thinness was between the global and national averages, while overweight and obesity rates remained below the 2011–2015 national levels [10]. Although regional heterogeneity in prevalence has been identified, large-scale, repeated studies tracking the evolving size and composition of the DBM in Xizang over the recent years remain scarce.

DBM is not the result of isolated factors but rather the product of a complex and dynamic interaction among multidimensional determinants, including individual biological behaviors, interpersonal family environments, community and school resources, macro-socioeconomics, and natural ecosystems [11,12]. Grounded in this Socio-Ecological Model (SEM) [13], we selected variables across individual, school, and macro-ecological tiers specifically tailored to the Xizang context: individual factors (e.g., dietary patterns [14,15], and physical activity levels [16]) govern physiological energy balance, school-level factors (e.g., school-based nutritional policies [17]) form the critical meso-level context shaping daily nutrient intake for this predominantly boarding population, and macro-level associated factors (e.g., socioeconomic growth [18], climate change [19] and altitude-latitude [7]) dictate regional food availability and environmental stress. In the unique context of Xizang, these multi-level factors may interact dynamically: the persistent risk of undernutrition may reflect the influence of high-altitude hypoxia and geographical constraints, whereas rapid local economic transitions and school-based dietary subsidies may increase energy availability, potentially contributing to a DBM landscape shaped by both persistent undernutrition and emerging overnutrition. While understanding of these independent correlates has matured, analyses that integrate multi-level associated factors in the specific context of high-altitude regions remain relatively rare.

Within this socio-ecological framework, the association of natural environmental factors with the nutritional status of this population remains controversial [20,21,22,23,24,25,26]. A cross-sectional study in Xizang from 1994 to 1995 argued that children’s nutritional status was unrelated to altitude [20], while an observational study conducted in Bolivia demonstrated a contrasting conclusion [21]. Regarding air quality, a study focusing on children and adolescents in Jiangsu Province indicated that ozone exposure was positively associated with the risk of overnutrition [23], which contrasts with the results of a nationwide cross-sectional study [24]. A study among Xizang children reported that long-term PM_2.5_ exposure was inversely associated with underweight but positively associated with stunting [25]. Nonetheless, a 2025 global ecological study yielded inconsistent conclusions [26]. The lack of consistent evidence hinders the optimization of resource allocation and policy formulation to address the double burden of malnutrition.

Utilizing data from five large-scale cross-sectional surveys conducted across Xizang from 2019 to 2024, this study operationally defines DBM at the population level to accurately describe the secular trends and inequalities in the population distribution of undernutrition and overnutrition among children and adolescents aged 6–17. Additionally, it seeks to identify the core correlates of the DBM at three levels: individual, school, and socio-ecological in the plateau region.

## 2. Materials and Methods

### 2.1. Study Population

This repeated cross-sectional study utilized the data from the National Common Diseases and Health Risk Factors Surveillance among Students in Xizang Autonomous Region of China from 2019 to 2024 (the surveillance was not conducted in 2022). Across all survey years, a standardized, multi-stage stratified random cluster sampling design was rigorously maintained by the same provincial CDC operation team, ensuring cross-year consistency and comparability of sampling procedures. The multi-stage sampling procedure was conducted as follows: In each county/district, the local CDC compiled a complete roster of all eligible schools, categorized them by school type, and assigned a unique sequential identification number to each school. Schools were then randomly selected using a simple random sampling method: in urban counties/districts, 2 primary schools, 2 middle schools, 2 regular senior high schools, and 1 vocational senior high school were chosen; in rural counties, 2 primary schools, 2 middle schools, and 1 regular senior high school were included. Within each selected school, classes in each grade were sequentially numbered, and two classes were randomly selected per grade using simple random sampling. All students enrolled in the selected classes were invited to participate as cluster subjects. If the combined number of students in the two selected classes within a grade was fewer than 80, additional classes were randomly selected from that grade following the same procedure until a minimum threshold of 80 students per grade was achieved.

Through physical examinations, a total of 311,119 students’ basic information and health status data were obtained. For the lifestyle questionnaire survey, all eligible students in Grade 4 and above (*N* = 191,927) were invited to participate. Physical examinations and lifestyle questionnaire surveys were administered as two relatively independent components within the surveillance program, and individual data were subsequently linked post-hoc using unique identification codes. Due to logistical constraints during field implementation, approximately 30% of records could not be validly matched (60,148 unmatched cases), resulting in 131,779 students with complete and validly matched physical examination and questionnaire data. Standardized Mean Differences (SMDs) were calculated to evaluate potential selection bias between students with matched questionnaire data and unmatched students, where an SMD < 0.10 indicates good balance between groups. Most baseline demographic and anthropometric characteristics, including age, gender, education level, residence type, and nutritional outcomes (undernutrition and overnutrition), showed high comparability between the matched questionnaire subsample and unmatched students, with all SMDs below 0.10 (Table S1). However, notable distribution differences were observed for altitude (SMD = 0.581) and socioeconomic status (SMD = 0.196), where the matched questionnaire subsample contained a higher proportion of students from lower-altitude (<3 km) and higher-SES areas.

**Figure 1:**
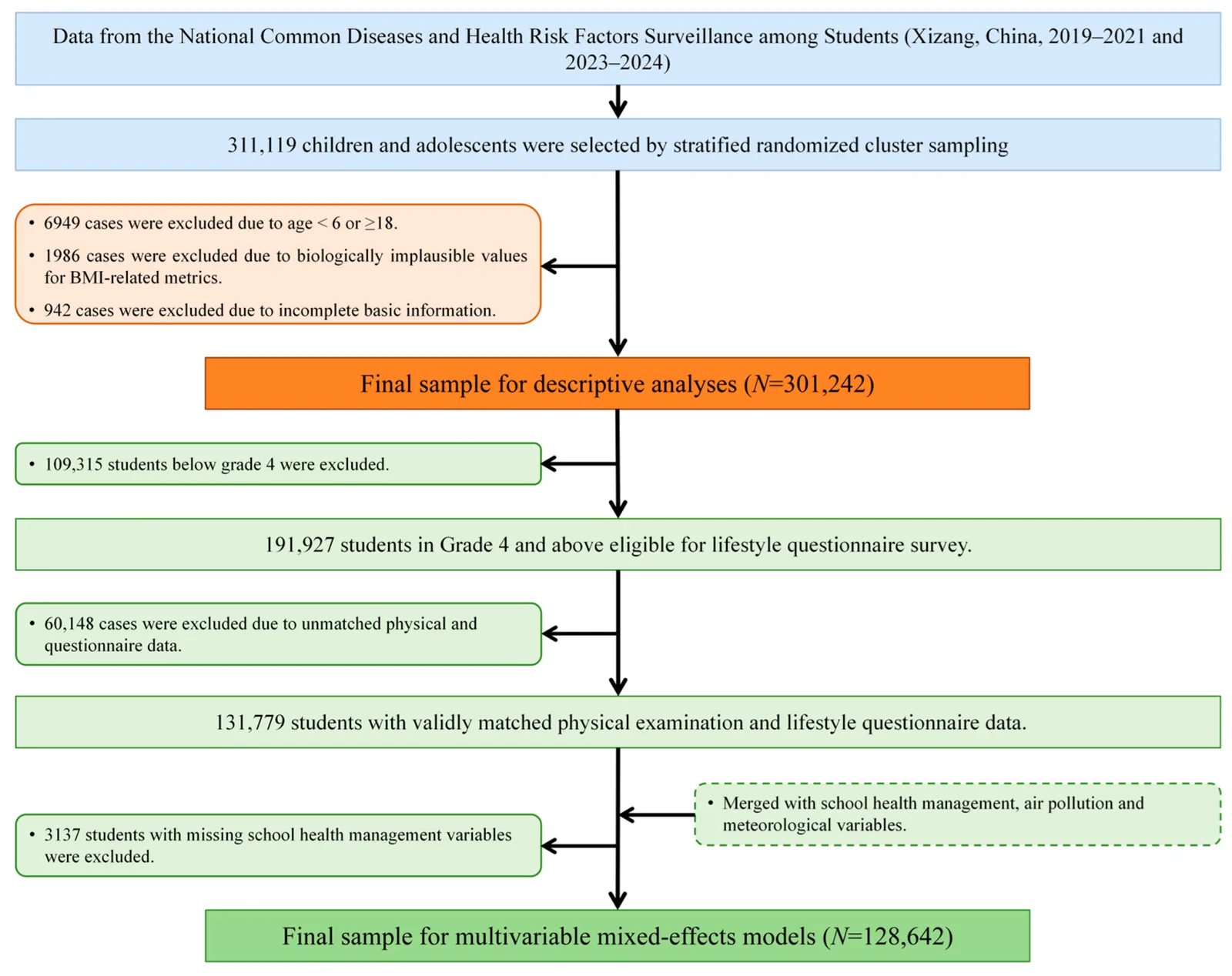
Flow chart of participants’ inclusion.

After excluding participants aged <6 or ≥18 years, those with missing basic information, and those with height or weight values exceeding ±5 standard deviations from the mean, a total of 301,242 participants were included in the analysis of nutritional status prevalence, and 128,642 participants were included in the association analysis (Figure 1).

### 2.2. Measures

#### 2.2.1. Double Burden of Malnutrition

In this study, DBM is operationalized at the population level as the coexistence and temporal trends of undernutrition and overnutrition within the study population, rather than an individual-level dual-phenotype. Height and weight were measured using standardized equipment. Z-scores for height-for-age (z-height) and BMI-for-age (z-BMI) were calculated using the official WHO AnthroPlus package (version 1.0.0), which implements the WHO 2007 Growth Reference for children and adolescents aged 5–19 years. Exact age in months was continuously derived from precise individual dates of birth and examination and directly entered into the AnthroPlus algorithm [27]. Undernutrition was defined as the presence of stunting (z-height < −2) or thinness (z-BMI < −2), while overnutrition was defined as the presence of overweight (1 < z-BMI ≤ 2) or obesity (z-BMI > 2) [27]. For regression modeling, undernutrition and overnutrition were analyzed as separate binary primary outcomes against normal nutritional status, with individuals exhibiting the alternative form of malnutrition excluded from the respective model. Notably, children exhibiting simultaneous stunting and overweight/obesity were retained and classified as overnutrition cases, as height deficits do not preclude excess weight-for-height.

#### 2.2.2. Lifestyle

Questionnaires were self-administered by the respondents. Intake of sugar-sweetened beverages (SSB) and fried foods was classified into “once a day or more” and “less than once a day”. Vegetables and fruit intake at least once per day was defined as sufficient [28]. Breakfast habit was classified into “every day” or “not every day”. Engaging in moderate-to-vigorous physical activity (MVPA) for ≥60 min per day was defined as sufficient [28].

#### 2.2.3. School Health Management

These indicators were reported by school administrators and linked to each respondent via a coding system. The indicators included: health education (launched or not), disease control (launched or not), exercises between classes (less than 5 times per week was defined as insufficient), annual sport meet (none, once, ≥2 times), school lunch menu (set or not), campus store (set or not).

#### 2.2.4. Socioeconomic Status

Socioeconomic status was determined based on the per capita Gross Domestic Product (GDP) of the county or district. Counties/districts ranked within the bottom 25% in that year were defined as less developed, those between 25% and 75% as moderately developed, and those above 75% as highly developed.

#### 2.2.5. Environmental and Meteorological Indicators

The location of each student was determined based on the city, district/county, and school name. The specific longitude and latitude of each school were obtained using the Amap web service [29]. Subsequently, in the R environment (version 4.4.0), point elevation values were extracted in batch for each coordinate using the get_elev_point() function from the elevatr package (version 0.99.1) [30], which queries the Amazon Web Services (AWS) Terrain Tiles digital elevation dataset. The extracted results were directly merged into the original survey database and analyzed in five categories: <3 km, 3–3.5 km, 3.5–4 km, 4–4.5 km, and ≥4.5 km.

Ambient air pollutant concentration data were derived from the Tracking Air Pollution (TAP) dataset [31,32,33]. Variables included in the analyses were particulate matter with an aerodynamic diameter ≤2.5 μm (PM_2.5_) and ozone. Monthly meteorological data were obtained from the ERA5-LAND reanalysis dataset released by the European Centre for Medium-Range Weather Forecasts (ECMWF) [34,35]. Variables included surface pressure, surface net solar radiation, 2 m temperature, and total precipitation. Matching of air pollutant and meteorological indicators was achieved by aligning longitude/latitude with the date of each student’s physical examination. The time window covered the month of examination and the preceding 11 months, and the 12-month average was calculated as the exposure data for each student.

#### 2.2.6. Covariates

Survey year, age (years), gender (male, female), education level (primary school, middle school, high school), residence (seat of the prefectural/municipal government was classified into urban, other urban districts/counties as town, and non-urban areas as rural), and family type (those living with at least one parent were defined as core family; others as non-core family) were included as covariates.

### 2.3. Mathematical Processing

Missing data in the exposures of interest, including lifestyle and school health management variables (Table S2), were addressed using a Random Forest multiple imputation approach (*m* = 5) via the mice package in R. Imputation was performed hierarchically according to the data structure: school health management variables were imputed at the school level using residence, county, altitude, survey year, and per capita GDP as predictors, and subsequently linked to individual records via unique school identifiers, whereas individual-level lifestyle variables were imputed using a set of demographic, geographical, socioeconomic, and outcome predictors, including age, gender, education level, residence, county, altitude, survey year, per capita GDP, undernutrition, and overnutrition.

Multicollinearity among independent variables was assessed across all five imputed datasets using variance inflation factors (VIFs). Given the high collinearity between surface pressure and altitude, surface pressure was excluded from the final regression models to ensure parsimony. Following its exclusion, all retained covariates exhibited mean equivalent VIFs well below the threshold of 5 (Table S2).

Given the repeated cross-sectional design, temporal dynamics were evaluated at the population level rather than tracking individuals longitudinally. In statistical modeling, survey year was incorporated both as a categorical variable (to estimate adjusted odds ratios for specific years relative to baseline) and as a continuous variable (to compute *P*_trend_ and assess linear temporal trends across the observation period).

### 2.4. Statistical Analysis

Categorical variables were presented as frequencies and percentages (%). For continuous variables, after testing for normality, those following a normal distribution were described as mean (standard deviation). Standardized Mean Differences (SMDs) were calculated for each covariate to assess the deviation in sample composition between different years. To account for potential variations in sample demographic composition across survey cycles, prevalence was standardized to the pooled study population using multivariable logistic regression adjusted for age, gender, education level, altitude, residence, and socioeconomic status, and standardized temporal trends were re-evaluated. For subgroup analyses, separate logistic regression models were fitted within strata of age, gender, education level, altitude, residence, and socioeconomic status, adjusting for the remaining covariates.

Two separate multivariable mixed-effects models (MMEMs) were constructed to examine the associations of individual, school, and social ecological level factors with undernutrition and overnutrition. School and county were incorporated as random intercepts to account for intra-cluster correlation. Every model was adjusted for the aforementioned covariates. All models were fitted separately across the 5 imputed datasets. Adjusted odds ratios (*aORs*), 95% confidence intervals (*CIs*), and *p*-values were pooled using Rubin’s rules to incorporate imputation-related uncertainty into the final inferences.

Two types of sensitivity analyses were performed. First, to assess the stability of temporal trends and minimize potential sampling bias from geographical variations, we restricted the sample to 10 districts/counties consistently surveyed across all five cycles (*n* = 80,137). Second, to evaluate the impact of missing data on the association models, the MMEMs were re-estimated using complete case analysis (CCA, *n* = 118,392), excluding any cases with missing values in the exposures of interest.

All analyses were conducted using R software (version 4.4.0). All statistical tests were two-sided, and the significance level was set at *p* < 0.05.

### 2.5. Ethical Considerations

The study was approved by the Medical Ethics Committee of the Xizang Center for Disease Control and Prevention (Approval Number: 2024-004, 29 September 2024). Given that this study involved secondary analysis of de-identified national surveillance data, the ethics committee granted a waiver of written informed consent. Per the national surveillance protocol, all participants and their legal guardians were verbally informed about the survey procedures, data usage, and their rights prior to participation.

## 3. Results

### 3.1. Basic Characteristics of Participants

As presented in Table 1, notable distributional imbalances across survey years were observed for age, education level, altitude, residence and socioeconomic status (all SMDs > 0.1). During this period, the proportion of high school students among the survey subjects, as well as the proportion of those living in urban and highly developed areas, gradually decreased. Most participants lived at altitudes between 3.5 km and 4.5 km.

### 3.2. Prevalence of DBM Changes from 2019 to 2024

**Figure 2:**
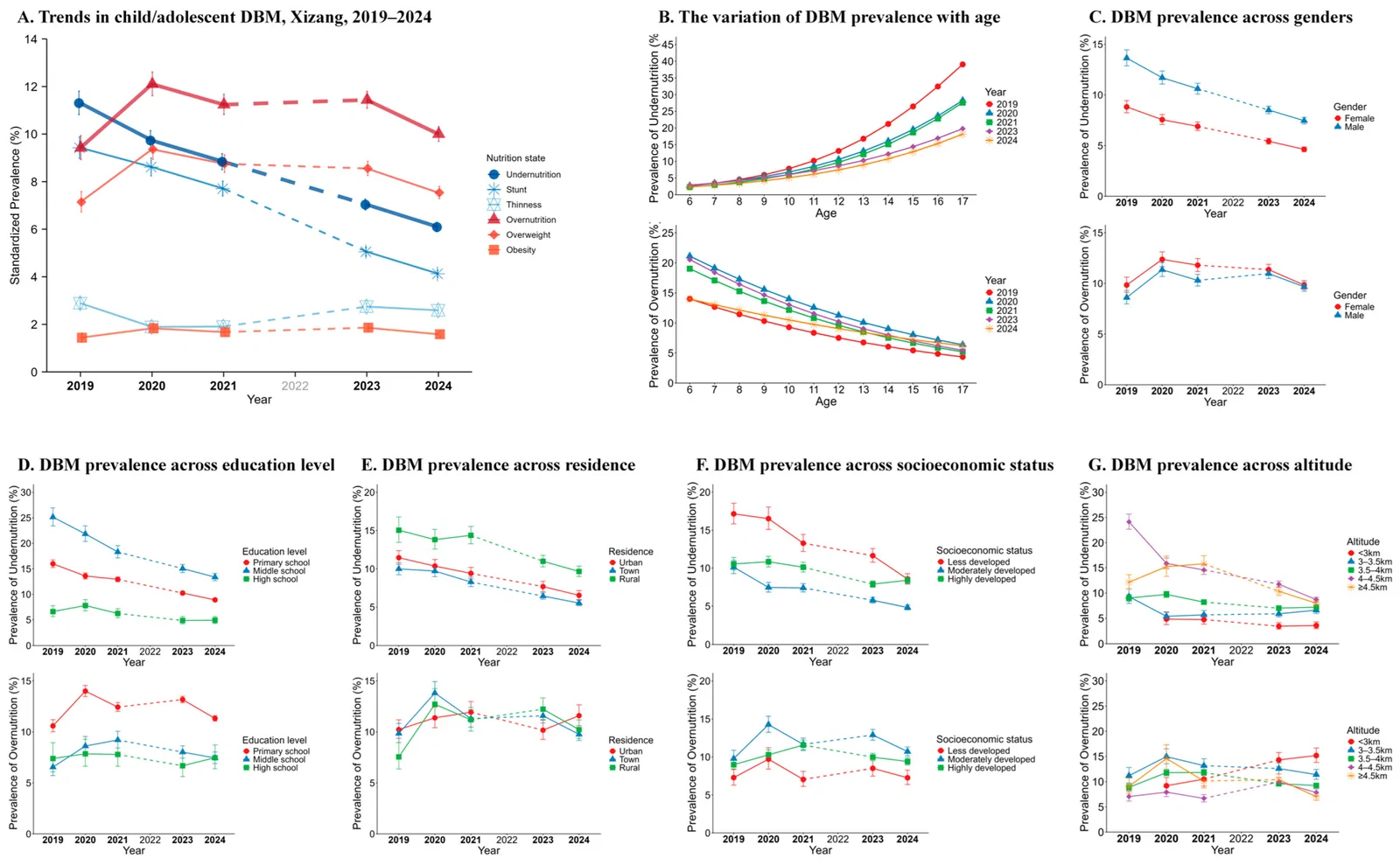
Trend of standardized prevalence of the DBM among children and adolescents in Xizang from 2019 to 2024 and subgroup differences. Note. Due to the absence of surveillance data in 2022, data points for 2021 and 2023 are connected by dashed lines for visual continuity only; this line does not represent an interpolated estimate for 2022.

To assess the population-level DBM, temporal trends were evaluated concurrently for both undernutrition and overnutrition (Figure 2A and Table S3)**.** The overall standardized prevalence of undernutrition decreased from 11.30% (95% *CI*: 10.81–11.81%) in 2019 to 9.73% (95% *CI*: 9.33–10.14%) in 2020, eventually reaching 6.09% (95% *CI*: 5.89–6.29%) by 2024. Stunting showed a consistent decline, falling from 9.42% (95% *CI*: 8.98–9.88%) in 2019 to 4.12% (95% *CI*: 3.98–4.28%) in 2024. Conversely, the standardized prevalence of thinness fluctuated; it dropped sharply to 1.89% in 2020 but subsequently rebounded to 2.75% in 2023, before slightly declining to 2.59% in 2024. The standardized prevalence of overnutrition peaked at 12.10% (95% *CI*: 11.61–12.61%) in 2020, up from 9.42% in 2019, before declining slightly to 11.24% (95% *CI*: 10.82–11.67%) in 2021. After a minor rebound to 11.44% (95% *CI*: 11.08–11.80%) in 2023, it dropped to 10.00% (95% *CI*: 9.69–10.32%) in 2024. Standardized prevalence for overweight and obesity exhibited trends similar to overall overnutrition, with 2024 levels remaining higher than those in 2019. Stunting served as the predominant component of undernutrition, whereas overweight constituted the primary contributor to overnutrition. Crude prevalence across survey cycles is detailed in Figure S1 and Tables S4–S6.

### 3.3. Subgroup Differences in the Prevalence of DBM

Figure 2B–G and Tables S7 and S8 present the standardized prevalence of DBM in different subgroups. With advancing age, the prevalence of undernutrition steadily increased, whereas overnutrition progressively declined (Figure 2B). Boys consistently exhibited a significantly higher standardized prevalence of undernutrition than girls, whereas overnutrition displayed the reverse trend (Figure 2C). By education level, middle school students had the highest prevalence of undernutrition, while primary school students bore the highest burden of overnutrition (Figure 2D). Geographically and economically, rural, less developed, and higher altitude areas had greater standardized prevalence of undernutrition (Figure 2E–G). In contrast, urban, moderately developed, and lower altitude regions had higher standardized prevalence of overnutrition. The standardized prevalence trends of DBM across different subgroups were largely consistent with those of the pooled sample. However, overnutrition in urban areas increased from 10.17% (95% *CI*: 9.26–11.16%) in 2023 to 11.59% (95% *CI*: 10.60–12.65%) in 2024, exhibiting a temporal trajectory contrary to that observed in other subgroups (Figure 2E). Meanwhile, the standardized prevalence of overnutrition in low-altitude areas (<3 km) showed a completely opposite trend compared to other altitude strata, continuously elevating from 9.19% (95% *CI*: 7.77–10.83%) in 2020 to 15.19% (95% *CI*: 13.81–16.67%) in 2024 (Figure 2G).

### 3.4. Association Between Exposures of Interest and DBM

**Figure 3:**
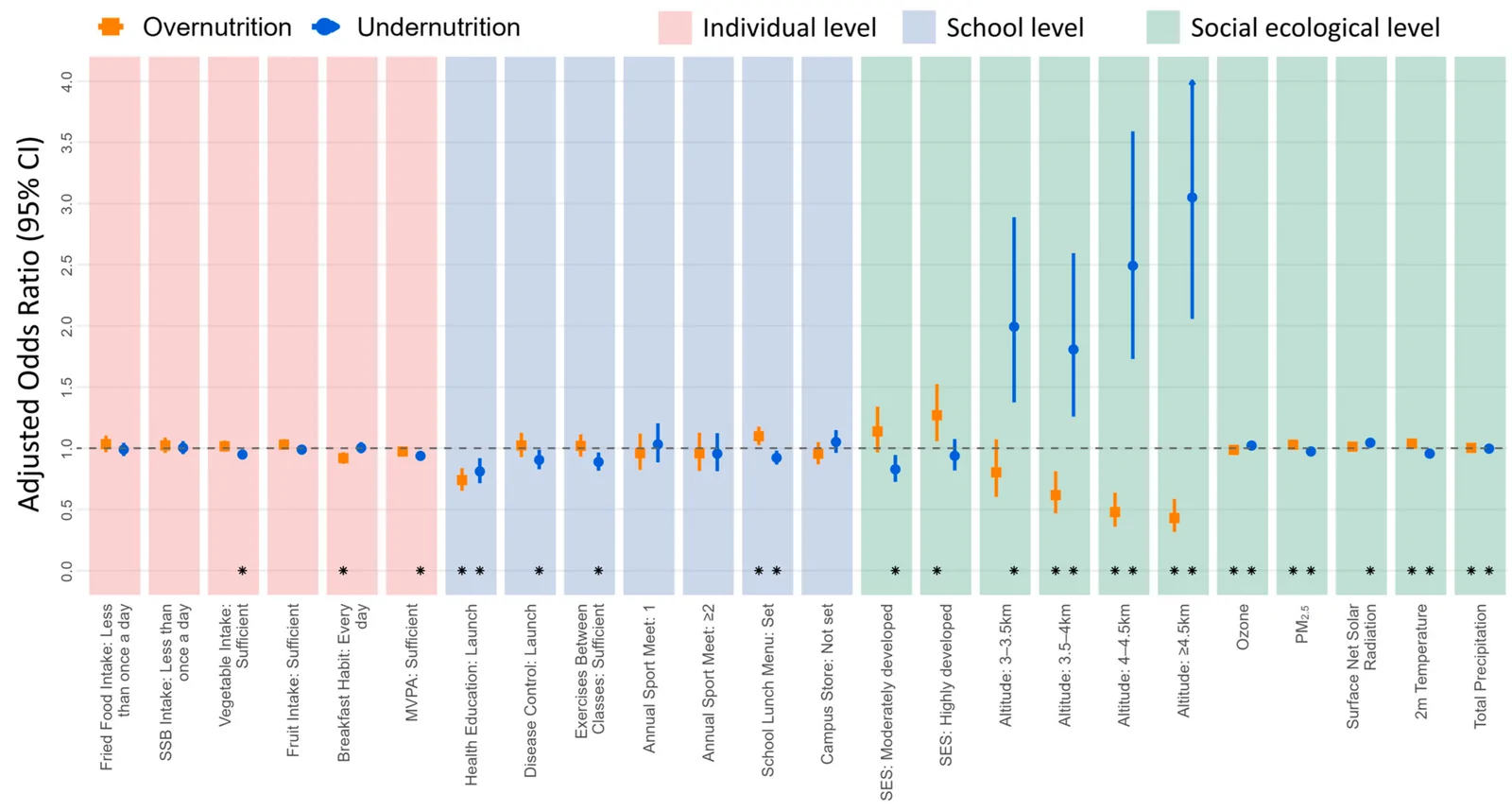
Association between exposures of interest and DBM. Note. School and county were included as random effects. Model adjusted for survey year, age, gender, education level, residence, and family type. Asterisk (*) indicates adjusted odds ratios whose 95% confidence interval do not include 1 (left: Overnutrition; right: Undernutrition). SSB: sugar-sweetened beverages. MVPA: moderate-to-vigorous physical activity. SES: socioeconomic status. PM_2.5_: particulate matter with an aerodynamic diameter ≤ 2.5 μm.

As presented in Figure 3 and Table S9, at the individual level, daily breakfast consumption was significantly negatively associated with the odds of overnutrition, with an *aOR* (95% *CI*) of 0.919 (0.873, 0.967). Sufficient vegetable intake and MVPA were notably associated with reduced odds of undernutrition, with *aORs* (95% *CI*) of 0.948 (0.913, 0.985) and 0.937 (0.904, 0.972), respectively.

At the school level, health education was associated with reduced odds of both undernutrition and overnutrition, with *aORs* (95% *CI*) of 0.810 (0.715, 0.918) and 0.739 (0.652, 0.838), respectively. Disease control measures, as well as exercises between classes five or more times per week, were associated with lower odds of undernutrition [*aORs* (95% *CI*) = 0.904 (0.828, 0.987) and 0.888 (0.817, 0.965) respectively]. The provision of school lunch menus was associated with reduced odds of undernutrition [*aOR* (95% *CI*) = 0.922 (0.867, 0.980)] and increased odds of overnutrition [*aOR* (95% *CI*) = 1.098 (1.024, 1.177)].

At the socio-ecological level, with “less developed regions” as the reference, children and adolescents in moderately developed regions exhibited significantly lower odds of undernutrition [*aOR* (95% *CI*) = 0.828 (0.725, 0.944)], while no statistically significant difference was observed for those in highly developed regions (*p* = 0.354). Conversely, residing in highly developed regions was associated with increased odds of overnutrition [*aOR* (95% *CI*) = 1.269 (1.056, 1.525)].

Compared with areas at an altitude of <3 km, those residing at altitudes of 3–3.5 km, 3.5–4 km, 4–4.5 km, and ≥4.5 km had increased odds of undernutrition [*aORs* (95% *CI*) = 1.993 (1.375, 2.888), 1.807 (1.259, 2.594), 2.491 (1.729, 3.588), and 3.050 (2.057, 4.521), respectively], while the odds of overnutrition decreased at altitudes of 3.5–4 km, 4–4.5 km, and ≥4.5 km [*aORs* (95% *CI*) = 0.616 (0.468, 0.813), 0.478 (0.359, 0.637), and 0.430 (0.315, 0.586), respectively].

Regarding air pollutants, elevated ozone concentrations were associated with increased odds of undernutrition [*aOR* (95% *CI*) = 1.021 (1.014, 1.029)] and reduced odds of overnutrition [*aOR* (95% *CI*) = 0.986 (0.979, 0.993)], whereas PM_2.5_ exhibited the opposite pattern [undernutrition: *aOR* (95% *CI*) = 0.973 (0.948, 0.999); overnutrition: *aOR* (95% *CI*) = 1.028 (1.001, 1.056)]. Among meteorological factors, increases in 2 m air temperature, and total precipitation were associated with decreased odds of undernutrition [*aORs* (95% *CI*) = 0.956 (0.933, 0.979) and 0.996 (0.994, 0.998), respectively], but were linked to elevated odds of overnutrition [*aORs* (95% *CI*) = 1.037 (1.016, 1.059) and 1.003 (1.001, 1.004), respectively]. Additionally, surface net solar radiation was significantly correlated with increased odds of undernutrition [*aOR* (95% *CI*) = 1.045 (1.014, 1.076)].

### 3.5. Sensitivity Analysis

The sensitivity analyses confirmed the robustness of both the temporal trends and the identified correlates. The observed prevalence trends in the 10 regions under continuous investigation (Figure S2 and Table S10) are highly consistent with the main analysis results. Regarding the factors associated with DBM, the results from the complete case analysis (Table S11) were mainly consistent with the primary model in terms of both the direction and magnitude of the adjusted odds ratios. Notably, marginal associations previously observed for PM_2.5_, 2 m air temperature, and total precipitation were no longer statistically significant.

## 4. Discussion

This study provides empirical evidence on the population-level double burden of malnutrition (DBM) among children and adolescents aged 6–17 years in Xizang from 2019 to 2024. Overall, the standardized prevalence of undernutrition decreased continuously from 11.30% to 6.09%, with stunting being the predominant form. Concurrently, the overnutrition prevalence peaked at 12.10% in 2020 and subsequently declined to 10.00% in 2024, remaining higher than the 2019 baseline (9.42%). Regarding association analysis, individual-level factors—including breakfast habits, fresh vegetable intake, and moderate-to-vigorous physical activity (MVPA)—were significantly associated with the odds of undernutrition and overnutrition, aligning with previous findings [14,15,16]. At the school level, health education, prevention and control of common diseases, frequency of exercises between classes, and implementation of school lunch menus showed significant associations with both forms of malnutrition to varying degrees. Consistent with existing domestic and international literature, socioeconomic development exhibited robust yet opposing associations with undernutrition versus overnutrition [18]. Finally, in terms of the natural environment, altitude, air pollutants, and meteorological factors were differentially associated with the two nutritional outcomes.

The crude prevalence of undernutrition among children and adolescents in Xizang is markedly higher than the national average [4], yet lower than that in nine low- and middle-income countries [36] and Nepal [37], which shares similar geographic conditions with Xizang. Conversely, the prevalence of overnutrition is lower than the national average [5], as well as lower than that of the United States [38], Nepal [37], and the East Asia and Pacific region [2] during the same period. These differences align with the gradient characteristics and association results of this study, which indicate that higher altitude correlates with an increased prevalence of undernutrition and a decreased prevalence of overnutrition. Contrasting with earlier conclusions that nutritional differences across altitudes are driven exclusively by social environments [20], our observational findings align with targeted studies in Bolivia [21] and Xizang [8] by demonstrating a persistent association between altitude gradients and nutritional outcomes. Rather than establishing a direct biological effect, altitude in our analyses serves as an integrative socio-ecological and geographical marker that reflects a complex interplay of environmental, behavioral, and structural factors. On one hand, external literature suggests that high-altitude exposure may influence nutritional dynamics through biological pathways such as altered resting metabolic rate [39], protein catabolism [40], and intestinal microbiota balance [41]—though these physiological parameters were not directly measured in the present survey. On the other hand, altitude is intrinsically tied to macro-level environmental and structural conditions, including socioeconomic development, agricultural yields, and dietary diversity, as well as potential sampling design shifts across survey cycles (such as the inclusion of populations below 3 km in later waves) [7]. Consequently, these associations warrant cautious interpretation and should not be construed as definitive proof of isolated biological causality.

Within the spectrum of undernutrition, children and adolescents in Xizang exhibit a unique pattern predominated by stunting, which stands in marked contrast to the thinness-predominated epidemiological pattern observed in other parts of China [4]. The results of this study show that between 2019 and 2024, the prevalence of stunting and thinness among Xizang children aged 6–17 years were 9.96% and 4.30%, respectively, yielding a stunting-to-thinness ratio of 2.32. This ratio is slightly lower than that of Nepal (2.59) [37], but notably higher than the 0.06 observed among Chinese Han students aged 7–18 years in 2019 [4] and the 1.13 reported across nine low- and middle-income countries from 2021 to 2023 [36]. This “stunting-thinness” inversion likely reflects multiple nutritional stress mechanisms unique to high-altitude regions. Specifically, the traditional dietary structure in Xizang’s agricultural and pastoral areas may be relatively deficient in high-quality protein and essential micronutrients, which constrains linear growth and leads to a high prevalence of stunting [42]. Furthermore, chronic high-altitude hypoxia shifts the action of insulin-like growth factor-1 (IGF-1) from promoting muscle and skeletal growth to merely maintaining survival through specific signaling pathways, thereby exacerbating the phenotypic expression of stunting [43]. Future nutritional interventions in Xizang should prioritize long-term, targeted supplementation of micronutrients and high-quality protein to effectively address the public health challenge centered on stunting.

While the global burden of overnutrition among children and adolescents is becoming increasingly severe, this study presents a starkly different landscape in Xizang. According to the GBD 2021 Adolescent BMI Collaborators, the prevalence of overweight and obesity has surged across all global regions since 1990, reaching 15.6% in the East Asia and Pacific region by 2021 [2]. In addition, results from the Chinese National Survey on Students’ Constitution and Health (CNSSCH) indicate that the prevalence of overnutrition among Chinese students aged 7–18 rose steadily from 1985 to 2019, and is projected to reach 47.8% by 2030 [5]. In contrast, the prevalence of overnutrition among children and adolescents in Xizang followed a downward trajectory after peaking in 2020. Although individual energy expenditure, ultra-processed food (UPF) consumption, and detailed dietary patterns were not directly measured in our study, this distinct trend can only be tentatively contextualized through plausible biological and structural hypotheses supported by external literature. Specifically, higher basal metabolic rates at high altitudes, coupled with daily physical exertion, have been hypothesized to constrain energy balance [39]. Furthermore, restricted market availability of UPFs and sugar-sweetened beverages, alongside traditional cereal- and dairy-based diets [42], represent potential, albeit unmeasured, structural explanations for mitigating overnutrition risk. Because these behavioral and environmental variables were not empirically assessed in our dataset, these mechanisms remain speculative and warrant further investigation in future prospective studies incorporating detailed dietary and physical activity assessments.

We observed that school-based health education was significantly associated with lower odds of both undernutrition and overnutrition, which is broadly consistent with previous research among Polish adolescents [44]. In regions where baseline nutritional literacy among students may be relatively limited, structured health education programs could play a vital role in supporting dietary awareness. Furthermore, health education may potentially operate synergistically with supportive school environments, such as the ongoing ‘Student Nutrition Improvement Program’ and ‘Nutritional Lunch’ initiatives in Xizang [45]. Classroom-learned dietary principles might be reinforced through nutritious school meals, serving as a hypothesized pathway toward balanced dietary habits. However, given the cross-sectional nature of our study, these associations do not establish causality. Potential confounding by indication—such as schools with greater nutritional challenges being prioritized for program implementation—cannot be ruled out. These proposed mechanisms should be interpreted as hypotheses for future prospective cohort or intervention studies.

In plateau regions where traditional dietary structures are relatively monotonous, the provision of school lunch menus was associated with lower odds of student undernutrition, a finding that aligns with previous research [17]. World Health Organization policy frameworks highlight school-based health and nutrition initiatives as potential levers for addressing the double burden of malnutrition [46]. The “Three Guarantees” policy (covering food, lodging, and education costs) and centralized meal programs implemented in Xizang may help address potential nutritional and energy gaps in household diets [9]. However, because our study evaluated school-level menu reporting rather than individual meal consumption, dietary quality, or program compliance, these observational findings cannot demonstrate whether school lunch programs directly reduce nutritional deficits. Notably, the provision of school lunch menus was also significantly associated with increased odds of overnutrition in our sample. Given our observational design, the temporal direction of this relationship cannot be established, and the potential for reverse causation (e.g., schools with higher baseline prevalence of overweight/obesity being more motivated to formalize lunch menus) or confounding by indication (e.g., menu programs being more readily implemented in economically better-off areas with higher overall energy intake) must be emphasized. This dual association highlights the complexity of school dietary environments in high-altitude regions and suggests that the mere presence of a lunch menu may not guarantee optimal dietary balance. Future longitudinal or interventional evaluations incorporating individual-level dietary intake are required to evaluate the effectiveness of school catering policies and inform more targeted school nutrition guidance.

Regarding air pollution, ozone was inversely associated with overnutrition and positively associated with undernutrition—findings aligned with a national cross-sectional study [24], though differing from observations in Jiangsu Province [23]. These discrepancies may stem from regional variations in geographical characteristics, climatic conditions, and pollutant concentration gradients. In addition, we observed marked ecological associations between meteorological factors and the double burden of malnutrition. While biological parameters and behavioral shifts were not directly measured in our study, these associations may reflect potential physiological or socioeconomic pathways. Specifically, existing literature suggests that milder climatic conditions might support physical development by mitigating chronic hypoxia or metabolic constraints at high altitudes, while environmental improvements often coincide with enhanced food accessibility and lifestyle transitions [47,48]. Notably, the attenuation observed in complete-case analysis suggests that the associations of PM_2.5_, temperature, and precipitation may be sensitive to missing-data handling and should not be regarded as robust independent correlates. Moreover, because environmental exposures were assigned using school-level annual averages rather than direct individual measurements, these observed ecological associations should be interpreted with caution and serve as hypothesis-generating finding for future mechanistic studies.

Our study possesses pronounced strengths, stemming from its comprehensive assessment of nutritional status using a large-scale, repeated dataset covering the entire Xizang Autonomous Region. By integrating multi-dimensional factors—ranging from individual behaviors and school-based health interventions to macro-socio-ecological factors—within a unified framework, this research provides novel insights into the complex mechanisms underlying the formation of DBM in extreme high-altitude environments.

However, several limitations must be acknowledged. First, the repeated cross-sectional design precludes the establishment of causal relationships. Second, data on students’ dietary behavior and physical activity were primarily derived from self-reported questionnaires, which may be subject to recall bias and social desirability bias. Third, field surveillance was suspended in 2022 due to regional COVID-19 lockdown restrictions in Xizang, resulting in a single-year data gap. Although trends could still be examined across the available pre- and post-2022 survey cycles, the absence of 2022 data limits the continuity of temporal assessment. Fourth, post-hoc record linkage yielded an unmatched rate of approximately 30% due to field constraints. While matched and unmatched groups were generally comparable, selection bias toward lower-altitude and higher-SES populations persisted. Despite analytical controls for missingness and sampling variation, residual selection bias and temporal compositional shifts warrant cautious interpretation. Fifth, unmeasured variables such as genetics and detailed home environment parameters were not incorporated, potentially leaving room for residual confounding. Sixth, environmental exposure data were assigned at the school level rather than through individual-level monitoring, which may introduce measurement errors in exposure–outcome estimates. Seventh, school-level variables relied on administrator reports and were uniformly assigned to students, reflecting policy environments rather than individual-level practices and potentially incurring exposure misclassification or ecological attribution bias. Finally, dietary habits were captured as dichotomous frequency categories based on the pre-designed national surveillance questionnaire, precluding granular dose–response assessment and potentially resulting in information loss.

## 5. Conclusions

In conclusion, repeated surveillance between 2019 and 2024 demonstrates a clear and sustained decline in undernutrition—particularly stunting—among children and adolescents in Xizang, while overnutrition followed a more complex trajectory, peaking in 2020 before declining yet remaining above the 2019 baseline in 2024. Furthermore, our findings identify significant multilevel associations across individual behaviors, school environments, and socio-ecological determinants. These observational associations should be interpreted as potential targets for future longitudinal investigations and intervention research, rather than demonstrated intervention pathways, when addressing the double burden of malnutrition in high-altitude contexts.

## Figures and Tables

**Table 1:** Basic characteristics of participants ^※^.

Variables	Overall (*N* = 301,242)	2019 (*n* = 17,413)	2020 (*n* = 25,340)	2021 (*n* = 36,725)	2023 (*n* = 95,141)	2024 (*n* = 126,623)	SMDs
Age (years), mean (SD)	11.09 (2.79)	11.45 (3.16)	11.54 (3.12)	11.28 (2.98)	10.95 (2.70)	11.00 (2.66)	0.111
Gender							0.006
Female	150,604 (49.99)	8720 (50.08)	12,722 (50.21)	18,535 (50.47)	47,588 (50.02)	63,039 (49.78)	
Male	150,638 (50.01)	8693 (49.92)	12,618 (49.79)	18,190 (49.53)	47,553 (49.98)	63,584 (50.22)	
Education level							0.227
Primary school	221,606 (73.56)	11,382 (65.36)	16,622 (65.60)	25,963 (70.70)	72,092 (75.77)	95,547 (75.46)	
Middle school	62,861 (20.87)	3690 (21.19)	5420 (21.39)	7150 (19.47)	19,717 (20.72)	26,884 (21.23)	
High school	16,775 (5.57)	2341 (13.44)	3298 (13.01)	3612 (9.84)	3332 (3.50)	4192 (3.31)	
Altitude							0.397
<3 km ^§^	14,893 (4.94)	—	1679 (6.63)	2232 (6.08)	4990 (5.24)	5992 (4.73)	
3~3.5 km	27,746 (9.21)	2407 (13.82)	3756 (14.82)	4144 (11.28)	8431 (8.86)	9008 (7.11)	
3.5~4 km	116,542 (38.69)	8070 (46.34)	12,751 (50.32)	17,805 (48.48)	34,057 (35.80)	43,859 (34.64)	
4~4.5 km	89,802 (29.81)	4509 (25.89)	5800 (22.89)	8828 (24.04)	29,308 (30.80)	41,357 (32.66)	
≥4.5 km	52,259 (17.35)	2427 (13.94)	1354 (5.34)	3716 (10.12)	18,355 (19.29)	26,407 (20.85)	
Residence							0.550
Urban	55,331 (18.37)	7848 (45.07)	11,789 (46.52)	12,801 (34.86)	11,137 (11.71)	11,756 (9.28)	
Town	144,196 (47.87)	6864 (39.42)	8398 (33.14)	14,651 (39.89)	47,542 (49.97)	66,741 (52.71)	
Rural	101,715 (33.77)	2701 (15.51)	5153 (20.34)	9273 (25.25)	36,462 (38.32)	48,126 (38.01)	
Socioeconomic status							0.363
Less developed	77,753 (25.81)	4261 (24.47)	4937 (19.48)	10,680 (29.08)	24,654 (25.91)	33,221 (26.24)	
Moderately developed	132,089 (43.85)	4885 (28.05)	7199 (28.41)	11,740 (31.97)	43,463 (45.68)	64,802 (51.18)	
Highly developed	91,400 (30.34)	8267 (47.48)	13,204 (52.11)	14,305 (38.95)	27,024 (28.40)	28,600 (22.59)	

^※^ Data for 2022 were unavailable as the surveillance was not conducted during that year. ^§^ All participants in 2019 resided in areas above an altitude of 3 km. SMD: standardized mean difference. SD: standard deviation.

## Data Availability

The datasets used and analyzed during the current study are available from the corresponding author on reasonable request.

## Supplementary Materials

The following supporting information can be downloaded at: https://www.mdpi.com/article/10.3390/nu18203340/s1, Figure S1: Trend of crude prevalence of the DBM among children and adolescents in Xizang from 2019 to 2024 and subgroup differences. Figure S2: Robustness analysis: Prevalence trends of undernutrition and overnutrition among children and adolescents in 10 consistently surveyed counties/districts from 2019 to 2024. Table S1: Comparison of baseline demographic and anthropometric characteristics between the questionnaire subsample and non-sampled students. Table S2: Multicollinearity diagnostics (Variance Inflation Factors, VIF) and variables missing conditions. Table S3: Standardized prevalence trends of undernutrition and overnutrition among children and adolescents in Xizang from 2019 to 2024 (%). Table S4: Prevalence trends of undernutrition and overnutrition among children and adolescents in Xizang from 2019 to 2024 (%). Table S5: Prevalence of the undernutrition among children and adolescents with different characteristics in Xizang from 2019 to 2024 (%). Table S6: Prevalence of the overnutrition among children and adolescents with different characteristics in Xizang from 2019 to 2024 (%). Table S7: Standardized prevalence of the undernutrition among children and adolescents with different characteristics in Xizang from 2019 to 2024 (%). Table S8: Standardized prevalence of the overnutrition among children and adolescents with different characteristics in Xizang from 2019 to 2024 (%). Table S9: Association between exposures of interest and DBM among children and adolescents in Xizang. Table S10: Robustness analysis: Prevalence trends of undernutrition and overnutrition among children and adolescents in 10 consistently surveyed counties/districts from 2019 to 2024 (%). Table S11: Robustness analysis: Association between exposures of interest and DBM among children and adolescents in Xizang using complete case analysis.
