# Supplementary material for: Epidemic Trends and Associated Factors of the Double Burden of Malnutrition Among Children and Adolescents in Xizang: Based on Five Cross-Sectional Surveys from 2019 to 2024
Source: Nutrients. 2026 Oct 12;18(20):3340. doi: 10.3390/nu18203340 (PMC13708046; doi:10.3390/nu18203340)
Supplement: Supplementary file 1 [file nutrients-18-03340-s001.zip › nutrients-4538010-supplementary.pdf]

Supplementary Materials

Dengcheng Zhang, Jianuo Jiang, Liming Tan, Zhuoma Pubu, Sujuan Li, Qiang Zhang, Li Chen, Jinghong Liang, Xinli Song, Yi Zhang, Ruolin Wang, Ziqi Dong, Yang Qin, Zhenting Lu, Jun Ma, Yanhui Dong.

Epidemic Trends and Associated Factors of the Double Burden of Malnutrition Among Children and Adolescents in Xizang: Based on Five Cross-Sectional Surveys from 2019 to 2024.

Figure S1: Trend of crude prevalence of the DBM among children and adolescents in Xizang from 2019 to 2024 and subgroup differences.

Figure S2: Robustness analysis: Prevalence trends of undernutrition and overnutrition among children and adolescents in 10 consistently surveyed counties/districts from 2019 to 2024.

Table S1: Comparison of baseline demographic and anthropometric characteristics between the questionnaire subsample and non-sampled students.

Table S2: Multicollinearity diagnostics (Variance Inflation Factors, VIF) and variables missing conditions.

Table S3: Standardized prevalence trends of undernutrition and overnutrition among children and adolescents in Xizang from 2019 to 2024 (%).

Table S4: Prevalence trends of undernutrition and overnutrition among children and adolescents in Xizang from 2019 to 2024 (%).

Table S5: Prevalence of the undernutrition among children and adolescents with different characteristics in Xizang from 2019 to 2024 (%).

Table S6: Prevalence of the overnutrition among children and adolescents with different characteristics in Xizang from 2019 to 2024 (%).

Table S7: Standardized prevalence of the undernutrition among children and adolescents with different characteristics in Xizang from 2019 to 2024 (%).

Table S8: Standardized prevalence of the overnutrition among children and adolescents with different characteristics in Xizang from 2019 to 2024 (%).

Table S9: Association between exposures of interest and DBM among children and adolescents in Xizang.

Table S10: Robustness analysis: Prevalence trends of undernutrition and overnutrition among children and adolescents in 10 consistently surveyed counties/districts from 2019 to 2024 (%).

Table S11: Robustness analysis: Association between exposures of interest and DBM among children and adolescents in Xizang using complete case analysis.

Figure S1: Trend of crude prevalence of the DBM among children and adolescents in Xizang from 2019 to 2024 and subgroup differences.

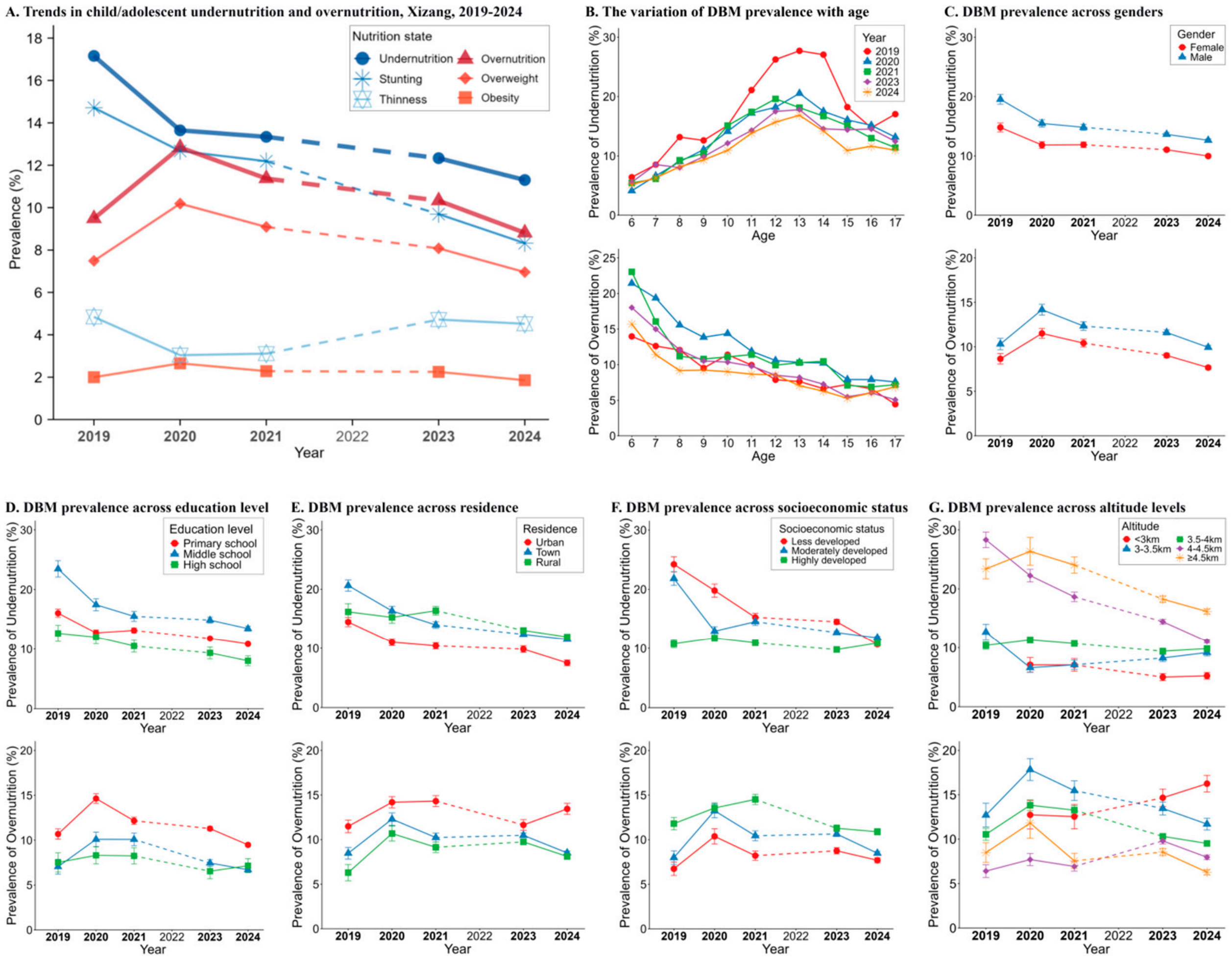

Note. Due to the absence of surveillance data in 2022, the prevalence of 2021 and 2023 are connected by a dashed line, which does not indicate an interpolated estimate for 2022.

Figure S2: Robustness analysis: Prevalence trends of undernutrition and overnutrition among children and adolescents in 10 consistently surveyed counties/districts from 2019 to 2024.

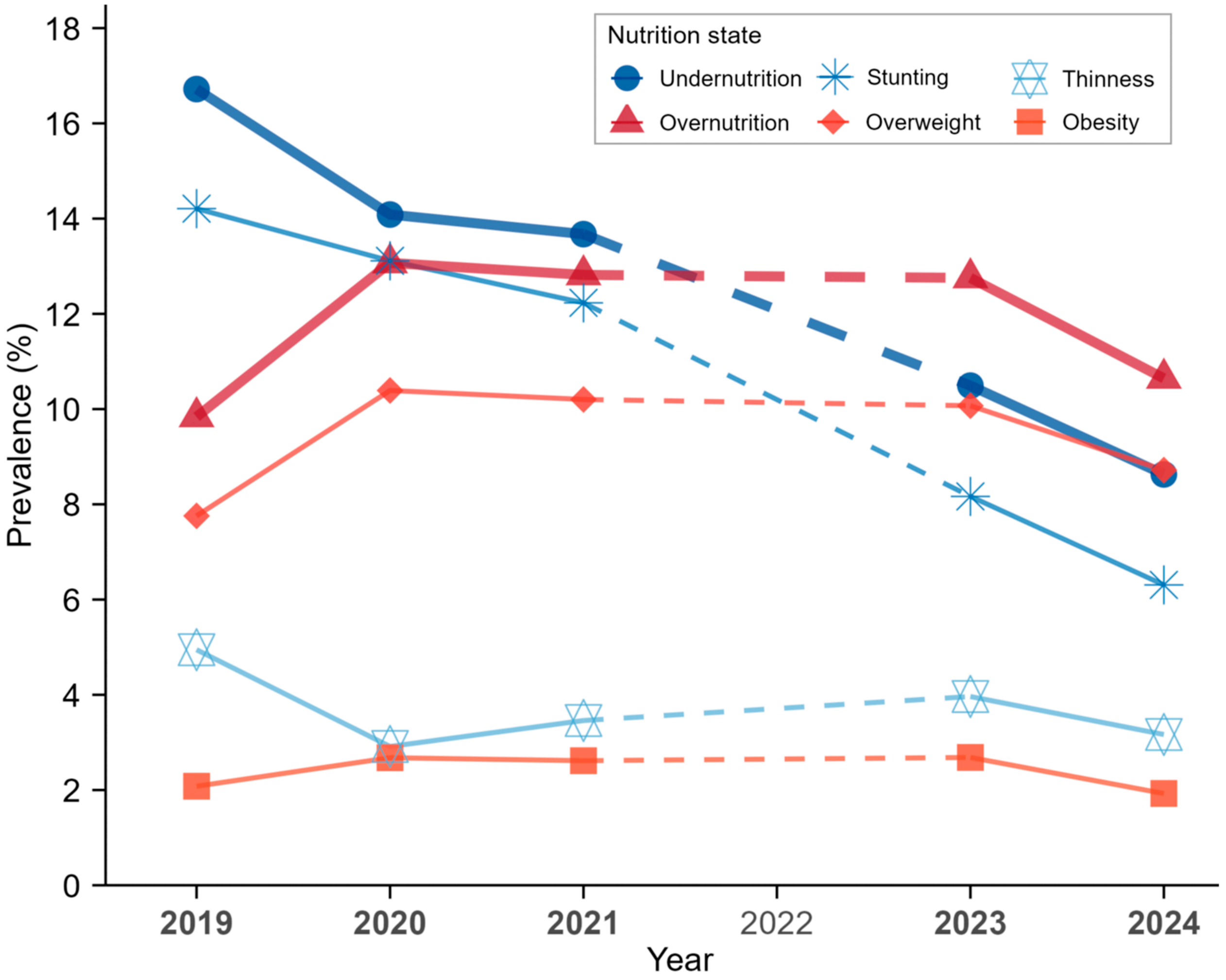

Note. Due to the absence of surveillance data in 2022, data points for 2021 and 2023 are connected by dashed lines for visual continuity only; this line does not represent an interpolated estimate for 2022.

Table S1: Comparison of baseline demographic and anthropometric characteristics between the questionnaire subsample and non-sampled students.

Variables	Questionnaire non-sampled (N = 60148)	Questionnaire subsample (N = 131779)	*P*	*SMD*
**Age(years), mean(SD)**	12.71 (2.05)	12.71 (2.12)	0.962	<0.001
**Gender**				
Female	30366 (50.5)	66218 (50.2)	0.340	0.005
Male	29782 (49.5)	65561 (49.8)		
**Education level**				
Primary school	35715 (59.4)	76576 (58.1)	<0.001	0.057
Middle school	19822 (33.0)	43039 (32.7)		
High school	4611 (7.7)	12164 (9.2)		
**Altitude**				
<3km	437 (0.7)	9373 (7.1)	<0.001	0.581
3-3.5km	2482 (4.1)	15395 (11.7)		
3.5-4km	21636 (36.0)	55166 (41.9)		
4-4.5km	19971 (33.2)	37110 (28.2)		
≥4.5km	15622 (26.0)	14735 (11.2)		
**Residence**				
Urban	12264 (20.4)	29916 (22.7)	<0.001	0.068
Town	32151 (53.5)	66364 (50.4)		
Rural	15733 (26.2)	35499 (26.9)		
**Socioeconomic status**				
Less developed	18375 (30.5)	29327 (22.3)	<0.001	0.196
Moderately developed	24877 (41.4)	57890 (43.9)		
Highly developed	16896 (28.1)	44562 (33.8)		
**BMI, mean(SD)**	17.92 (2.60)	17.99 (2.69)	<0.001	0.029
**Undernutrition**				
No	45475 (82.5)	101921 (85.0)	<0.001	0.068
Yes	9636 (17.5)	17962 (15.0)		
**Stunting**				
No	52396 (87.1)	118200 (89.7)	<0.001	0.081
Yes	7752 (12.9)	13579 (10.3)		
**Thinness**				
No	57024 (94.8)	125251 (95.0)	0.026	0.011
Yes	3124 (5.2)	6528 (5.0)		
**Overnutrition**				
No	51987 (91.2)	113355 (90.5)	<0.001	0.023
Yes	5037 (8.8)	11896 (9.5)		
**Overweight**				
No	55867 (92.9)	121823 (92.4)	0.001	0.017
Yes	4281 (7.1)	9956 (7.6)		
**Obesity**				
No	59392 (98.7)	129839 (98.5)	<0.001	0.019
Yes	756 (1.3)	1940 (1.5)		

Table S2: Multicollinearity diagnostics (Variance Inflation Factors, VIF) and variables missing conditions.

Variable	VIFs before exclusion of Surface pressure			VIFs after exclusion of Surface pressure			Missing (%)
	Mean	Min	Max	Mean	Min	Max	
Surface pressure	7.84	7.82	7.86	—	—	—	—
Altitude	7.52	7.50	7.53	4.15	4.15	4.16	—
2m temperature	5.12	5.12	5.12	3.62	3.61	3.62	—
Age	3.84	3.84	3.84	3.85	3.85	3.85	—
Ozone	3.81	3.80	3.83	3.78	3.77	3.80	—
Surface net solar radiation	3.22	3.22	3.22	2.99	2.98	2.99	—
Education level	2.39	2.39	2.40	2.40	2.40	2.40	—
Total precipitation	2.01	2.00	2.01	1.96	1.96	1.97	—
Residence	1.72	1.71	1.72	1.71	1.70	1.71	—
$PM_{2.5}$	1.59	1.59	1.59	1.57	1.57	1.57	—
Presence of a campus store	1.52	1.51	1.54	1.54	1.52	1.56	2.73
Socioeconomic status	1.43	1.43	1.43	1.42	1.42	1.42	—
Fruit intake	1.23	1.23	1.23	1.23	1.23	1.23	1.88
Vegetable intake	1.21	1.21	1.21	1.21	1.21	1.21	1.89
Sugar-sweetened beverage intake	1.17	1.17	1.17	1.17	1.17	1.17	1.87
Fried food intake	1.16	1.16	1.16	1.16	1.16	1.16	1.87
School lunch menu	1.10	1.09	1.11	1.09	1.08	1.10	5.59
Annual sport meet	1.10	1.09	1.10	1.09	1.09	1.10	2.93
Disease control	1.09	1.09	1.09	1.09	1.09	1.09	2.31
Exercises between classes	1.07	1.07	1.07	1.06	1.06	1.07	2.84
Health education	1.06	1.06	1.07	1.06	1.06	1.07	2.17
Breakfast habit	1.06	1.06	1.06	1.06	1.06	1.06	1.88
Moderate-to-high-intensity exercise	1.05	1.05	1.05	1.05	1.05	1.05	2.05
Family type	1.02	1.02	1.02	1.02	1.02	1.02	2.20
Gender	1.01	1.01	1.01	1.01	1.01	1.01	—

Table S3: Standardized prevalence trends of undernutrition and overnutrition among children and adolescents in Xizang from 2019 to 2024 (%).

Outcome	Standardized prevalence (95% *CI*)					
	2019	2020	2021	2023	2024	*P* for trend
Undernutrition	11.30 (10.81, 11.81)	9.73 (9.33, 10.14)	8.83 (8.50, 9.18)	7.03 (6.81, 7.27)	6.09 (5.89, 6.29)	＜0.001
Stunting	9.42 (8.98, 9.88)	8.61 (8.24, 9.00)	7.70 (7.40, 8.02)	5.05 (4.87, 5.24)	4.12 (3.98, 4.28)	＜0.001
Thinness	2.89 (2.66, 3.13)	1.89 (1.74, 2.06)	1.91 (1.77, 2.05)	2.75 (2.60, 2.90)	2.59 (2.46, 2.73)	＜0.001
Overnutrition	9.42 (8.93, 9.93)	12.10 (11.61, 12.61)	11.24 (10.82, 11.67)	11.44 (11.08, 11.80)	10.00 (9.69, 10.32)	＜0.001
Overweight	7.14 (6.73, 7.58)	9.36 (8.94, 9.81)	8.75 (8.39, 9.13)	8.55 (8.26, 8.86)	7.54 (7.28, 7.80)	＜0.001
Obesity	1.44 (1.26, 1.64)	1.83 (1.64, 2.04)	1.67 (1.51, 1.85)	1.86 (1.70, 2.03)	1.58 (1.45, 1.72)	0.670

Note. No data available for 2022. *CI*: Confidence interval. Prevalence was standardized to the pooled study population using multivariable logistic regression adjusted for age, gender, education level, altitude, residence, and socioeconomic status.

Table S4: Prevalence trends of undernutrition and overnutrition among children and adolescents in Xizang from 2019 to 2024 (%).

Outcome	Prevalence (95% *CI*)					
	**Overall**	**2019**	**2020**	**2021**	**2023**	**2024**
Undernutrition	12.41 (12.30, 12.53)	17.16 (16.61, 17.73)	**13.65* (13.23, 14.07)**	13.34 (13.00, 13.69)	**12.33* (12.13, 12.54)**	**11.30* (11.13, 11.48)**
Stunting	9.96 (9.86, 10.07)	14.71 (14.19, 15.25)	**12.67* (12.27, 13.09)**	12.19 (11.86, 12.53)	**9.69* (9.51, 9.88)**	**8.32* (8.17, 8.48)**
Thinness	4.30 (4.23, 4.38)	4.84 (4.53, 5.17)	**3.04* (2.83, 3.26)**	3.11 (2.94, 3.29)	**4.72* (4.58, 4.85)**	**4.52* (4.41, 4.64)**
Overnutrition	9.98 (9.87, 10.09)	9.49 (9.06, 9.93)	**12.84* (12.44, 13.26)**	**11.37* (11.05, 11.70)**	**10.33* (10.14, 10.52)**	**8.81* (8.66, 8.97)**
Overweight	7.87 (7.78, 7.97)	7.49 (7.11, 7.89)	**10.19* (9.82, 10.57)**	**9.09* (8.80, 9.39)**	**8.08* (7.91, 8.25)**	**6.96* (6.82, 7.10)**
Obesity	2.11 (2.06, 2.16)	2.00 (1.80, 2.22)	**2.65* (2.46, 2.86)**	**2.28* (2.14, 2.44)**	2.25 (2.16, 2.35)	**1.86* (1.78, 1.93)**

Note. No data available for 2022. *CI*: Confidence interval. Chi-square tests were used to compare differences in DBM prevalence between two consecutive surveys. Bold and * indicate that there is a statistically significant difference in prevalence compared to the previous survey.

Table S5: Prevalence of the undernutrition among children and adolescents with different characteristics in Xizang from 2019 to 2024 (%).

Groups	Total	2019	2020	2021	2023	2024
Gender						
Male	13.85 (13.67, 14.02)	19.53 (18.71, 20.38)	**15.47 * (14.85, 16.11)**	**14.82 * (14.31, 15.35)**	**13.63 * (13.33, 13.94)**	**12.63 * (12.37, 12.89)**
Female	10.98 (10.82, 11.14)	14.79 (14.06, 15.55)	**11.84 * (11.29, 12.41)**	11.89 (11.43, 12.36)	**11.04 * (10.76, 11.32)**	**9.97 * (9.74, 10.20)**
Education level						
Primary school	11.83 (11.69, 11.96)	16.04 (15.38, 16.73)	**12.73 * (12.23, 13.25)**	13.14 (12.73, 13.55)	**11.78 * (11.54, 12.02)**	**10.84 * (10.65, 11.04)**
Middle school	15.06 (14.79, 15.35)	23.47 (22.13, 24.86)	**17.45 * (16.47, 18.49)**	**15.51 * (14.69, 16.37)**	**14.88 * (14.39, 15.38)**	**13.45 * (13.04, 13.86)**
High school	10.24 (9.79, 10.71)	12.64 (11.36, 14.05)	12.01 (10.94, 13.16)	**10.49 * (9.54, 11.53)**	9.33 (8.39, 10.37)	8.02 (7.23, 8.88)
Residence						
Urban	10.39 (10.14, 10.65)	14.46 (13.70, 15.26)	**11.03 * (10.47, 11.61)**	10.41 (9.90, 10.95)	**9.85 * (9.31, 10.42)**	**7.53 * (7.06, 8.02)**
Town	12.76 (12.59, 12.94)	20.63 (19.69, 21.60)	**16.34 * (15.56, 17.14)**	**13.98 * (13.43, 14.55)**	**12.37 * (12.08, 12.67)**	**11.52 * (11.28, 11.76)**
Rural	13.01 (12.81, 13.22)	16.18 (14.84, 17.62)	15.25 (14.30, 16.26)	16.37 (15.63, 17.14)	**13.04 * (12.70, 13.39)**	**11.93 * (11.64, 12.22)**
Socioeconomic status						
Less developed	13.85 (13.60, 14.09)	24.22 (22.96, 25.53)	**19.79 * (18.70, 20.92)**	**15.26 * (14.59, 15.96)**	14.51 (14.08, 14.96)	**10.68 * (10.35, 11.02)**
Moderately developed	12.77 (12.59, 12.95)	21.82 (20.69, 23.00)	**12.92 * (12.16, 13.71)**	**14.53 * (13.91, 15.18)**	**12.68 * (12.37, 13.00)**	**11.80 * (11.56, 12.05)**
Highly developed	10.68 (10.48, 10.88)	10.77 (10.12, 11.45)	**11.75 * (11.21, 12.31)**	10.93 (10.43, 11.45)	**9.79 * (9.44, 10.15)**	**10.89 * (10.54, 11.26)**
Altitude						
<3km†	5.64 (5.28, 6.02)	—	7.09 (5.96, 8.42)	7.08 (6.09, 8.22)	**5.01 * (4.44, 5.65)**	5.22 (4.69, 5.82)
3-3.5km	8.53 (8.21, 8.87)	12.67 (11.40, 14.06)	**6.60 * (5.85, 7.44)**	7.09 (6.35, 7.92)	8.24 (7.68, 8.85)	9.17 (8.59, 9.78)
3.5-4km	10.04 (9.87, 10.22)	10.38 (9.74, 11.07)	**11.32 * (10.78, 11.88)**	10.70 (10.26, 11.17)	**9.40 * (9.10, 9.72)**	9.84 (9.56, 10.12)
4-4.5km	14.51 (14.28, 14.74)	28.32 (27.02, 29.65)	**22.26 * (21.21, 23.35)**	**18.66 * (17.86, 19.48)**	**14.44 * (14.05, 14.85)**	**11.09 * (10.79, 11.39)**
≥4.5km	18.08 (17.75, 18.41)	23.40 (21.76, 25.13)	**26.37 * (24.09, 28.78)**	**24.06 * (22.71, 25.46)**	**18.27 * (17.72, 18.84)**	**16.18 * (15.74, 16.63)**

Note. No data available for 2022. *CI*: Confidence interval. Chi-square tests were used to compare differences in DBM prevalence between two consecutive surveys. Bold and * indicate that there is a statistically significant difference in prevalence compared to the previous survey. † All participants in 2019 resided in areas above an altitude of 3 kilometers.

Table S6: Prevalence of the overnutrition among children and adolescents with different characteristics in Xizang from 2019 to 2024 (%).

Groups	Total	2019	2020	2021	2023	2024
Gender						
Male	11.14 (10.98, 11.30)	10.32 (9.70, 10.98)	**14.18 * (13.58, 14.80)**	**12.34 * (11.87, 12.83)**	11.62 (11.33, 11.91)	**9.94 * (9.71, 10.18)**
Female	8.82 (8.68, 8.97)	8.66 (8.09, 9.27)	**11.52 * (10.97, 12.08)**	**10.42 * (9.99, 10.87)**	**9.04 * (8.78, 9.30)**	**7.67 * (7.47, 7.88)**
Education level						
Primary school	10.83 (10.70, 10.96)	10.68 (10.13, 11.26)	**14.64 * (14.11, 15.18)**	**12.16 * (11.77, 12.57)**	**11.29 * (11.06, 11.52)**	**9.48 * (9.29, 9.66)**
Middle school	7.64 (7.43, 7.85)	7.05 (6.26, 7.92)	**10.09 * (9.32, 10.92)**	10.08 (9.41, 10.80)	**7.45 * (7.09, 7.83)**	**6.71 * (6.41, 7.01)**
High school	7.55 (7.16, 7.96)	7.52 (6.52, 8.66)	8.31 (7.41, 9.30)	8.25 (7.40, 9.19)	**6.54 * (5.75, 7.43)**	7.16 (6.41, 7.98)
Residence						
Urban	13.16 (12.88, 13.44)	11.48 (10.79, 12.20)	**14.18 * (13.56, 14.82)**	14.31 (13.72, 14.93)	**11.64 * (11.05, 12.25)**	**13.44 * (12.84, 14.07)**
Town	9.55 (9.40, 9.70)	8.46 (7.83, 9.15)	**12.29 * (11.60, 13.01)**	**10.22 * (9.74, 10.73)**	10.47 (10.20, 10.75)	**8.51 * (8.30, 8.72)**
Rural	8.87 (8.69, 9.04)	6.29 (5.44, 7.27)	**10.67 * (9.86, 11.55)**	**9.13 * (8.56, 9.74)**	**9.74 * (9.44, 10.05)**	**8.11 * (7.87, 8.35)**
Socioeconomic status						
Less developed	8.21 (8.02, 8.41)	6.74 (6.02, 7.53)	**10.37 * (9.55, 11.25)**	**8.20 * (7.70, 8.74)**	**8.75 * (8.40, 9.11)**	**7.69 * (7.41, 7.98)**
Moderately developed	9.60 (9.44, 9.76)	7.98 (7.26, 8.78)	**13.24 * (12.47, 14.04)**	**10.43 * (9.89, 11.00)**	10.63 (10.35, 10.93)	**8.47 * (8.26, 8.69)**
Highly developed	12.04 (11.83, 12.25)	11.79 (11.12, 12.51)	**13.55 * (12.98, 14.14)**	**14.51 * (13.94, 15.10)**	**11.28 * (10.90, 11.66)**	10.88 (10.53, 11.25)
Altitude						
<3km†	14.77 (14.21, 15.35)	—	12.75 (11.24, 14.43)	12.54 (11.23, 13.98)	**14.67 * (13.71, 15.68)**	**16.26 * (15.34, 17.21)**
3-3.5km	13.72 (13.32, 14.13)	12.71 (11.44, 14.10)	**17.84 * (16.65, 19.10)**	**15.47 * (14.40, 16.60)**	**13.46 * (12.75, 14.21)**	**11.70 * (11.05, 12.38)**
3.5-4km	10.87 (10.69, 11.05)	10.55 (9.89, 11.23)	**13.83 * (13.24, 14.44)**	13.27 (12.78, 13.78)	**10.32 * (10.00, 10.65)**	**9.52 * (9.25, 9.80)**
4-4.5km	8.37 (8.19, 8.55)	6.41 (5.73, 7.16)	**7.71 * (7.05, 8.42)**	6.93 (6.42, 7.48)	**9.81 * (9.47, 10.16)**	**7.96 * (7.71, 8.23)**
≥4.5km	7.42 (7.20, 7.64)	8.49 (7.44, 9.66)	**11.82 * (10.20, 13.65)**	**7.56 * (6.75, 8.46)**	8.55 (8.16, 8.97)	**6.28 * (6.00, 6.58)**

Note. No data available for 2022. *CI*: Confidence interval. Chi-square tests were used to compare differences in DBM prevalence between two consecutive surveys. Bold and * indicate that there is a statistically significant difference in prevalence compared to the previous survey. † All participants in 2019 resided in areas above an altitude of 3 kilometers.

Table S7: Standardized prevalence of the undernutrition among children and adolescents with different characteristics in Xizang from 2019 to 2024 (%).

Groups	2019	2020	2021	2023	2024	*P* for trend
Gender						
Female	8.82 (8.24, 9.44)	**7.56 * (7.09, 8.07)**	**6.90 * (6.50, 7.32)**	**5.43 * (5.16, 5.72)**	**4.63 * (4.40, 4.87)**	<0.001
Male	13.64 (12.87, 14.45)	**11.70 * (11.07, 12.36)**	**10.61 * (10.08, 11.15)**	**8.51 * (8.15, 8.89)**	**7.46 * (7.14, 7.78)**	<0.001
Education level						
Primary school	15.97 (15.24, 16.73)	**13.58 * (13.00, 14.18)**	12.93 (12.47, 13.40)	**10.24 * (9.95, 10.54)**	**8.90 * (8.65, 9.16)**	<0.001
Middle school	25.13 (23.40, 26.94)	**21.83 * (20.35, 23.40)**	**18.30 * (17.13, 19.54)**	**15.04 * (14.26, 15.85)**	**13.36 * (12.65, 14.10)**	<0.001
High school	6.61 (5.62, 7.76)	7.79 (6.76, 8.96)	**6.24 * (5.41, 7.18)**	**4.86 * (4.18, 5.64)**	4.91 (4.26, 5.65)	<0.001
Residence						
Urban	11.45 (10.58, 12.37)	10.35 (9.53, 11.22)	9.40 (8.68, 10.18)	**7.67 * (7.02, 8.38)**	**6.53 * (5.96, 7.16)**	<0.001
Town	9.98 (9.22, 10.79)	9.70 (8.96, 10.49)	**8.27 * (7.67, 8.91)**	**6.43 * (6.02, 6.86)**	**5.52 * (5.17, 5.90)**	<0.001
Rural	15.04 (13.45, 16.77)	13.81 (12.58, 15.15)	14.37 (13.27, 15.54)	**10.98 * (10.25, 11.76)**	**9.65 * (8.99, 10.35)**	<0.001
Socioeconomic status						
Less developed	17.14 (15.83, 18.54)	16.51 (15.09, 18.04)	**13.28 * (12.21, 14.43)**	**11.64 * (10.77, 12.57)**	**8.56 * (7.90, 9.27)**	<0.001
Moderately developed	10.13 (9.29, 11.04)	**7.46 * (6.85, 8.13)**	7.40 (6.87, 7.97)	**5.77 * (5.40, 6.16)**	**4.82 * (4.51, 5.14)**	<0.001
Highly developed	10.55 (9.75, 11.40)	10.84 (10.16, 11.55)	10.12 (9.49, 10.78)	**7.89 * (7.47, 8.32)**	8.41 (7.98, 8.87)	<0.001
Altitude						
<3km†	—	4.83 (3.75, 6.18)	4.74 (3.83, 5.86)	**3.42 * (2.84, 4.12)**	3.56 (2.97, 4.27)	0.003
3-3.5km	9.29 (7.94, 10.84)	**5.38 * (4.61, 6.27)**	5.67 (4.92, 6.54)	5.89 (5.25, 6.60)	6.60 (5.94, 7.34)	0.236
3.5-4km	9.02 (8.37, 9.73)	9.73 (9.14, 10.35)	**8.20 * (7.75, 8.68)**	**7.01 * (6.65, 7.37)**	7.21 (6.88, 7.56)	<0.001
4-4.5km	24.13 (22.66, 25.67)	**15.88 * (14.85, 16.97)**	14.59 (13.70, 15.53)	**11.76 * (11.15, 12.39)**	**8.70 * (8.24, 9.18)**	<0.001
≥4.5km	12.15 (10.80, 13.64)	**15.23 * (13.33, 17.35)**	15.81 (14.35, 17.38)	**10.35 * (9.52, 11.24)**	**7.98 * (7.32, 8.69)**	<0.001

Note. No data available for 2022. *CI*: Confidence interval. Bold and * indicate that there is a statistically significant difference in prevalence compared to the previous survey. † All participants in 2019 resided in areas above an altitude of 3 kilometers. Separate logistic regression models were fitted to assess subgroup standardized prevalence within strata of age, gender, school level, altitude, residence, and socioeconomic status, adjusting for the remaining covariates.

Table S8: Standardized prevalence of the overnutrition among children and adolescents with different characteristics in Xizang from 2019 to 2024 (%).

Groups	2019	2020	2021	2023	2024	*P* for trend
Gender						
Female	9.84 (9.12, 10.62)	**12.37 * (11.66, 13.12)**	11.80 (11.18, 12.45)	11.36 (10.87, 11.88)	**9.84 * (9.41, 10.28)**	<0.001
Male	8.61 (7.98, 9.29)	**11.35 * (10.69, 12.05)**	**10.30 * (9.74, 10.89)**	**10.97 * (10.48, 11.49)**	**9.67 * (9.24, 10.12)**	0.244
Education level						
Primary school	10.59 (10.02, 11.20)	**14.00 * (13.46, 14.55)**	**12.43 * (12.01, 12.87)**	**13.17 * (12.86, 13.49)**	**11.32 * (11.06, 11.59)**	<0.001
Middle school	6.55 (5.72, 7.49)	**8.61 * (7.75, 9.55)**	9.21 (8.42, 10.06)	**8.02 * (7.43, 8.64)**	7.45 (6.90, 8.03)	0.069
High school	7.39 (6.09, 8.94)	7.85 (6.63, 9.28)	7.79 (6.63, 9.14)	6.68 (5.61, 7.94)	7.47 (6.39, 8.72)	0.399
Residence						
Urban	10.23 (9.36, 11.18)	11.38 (10.41, 12.43)	11.93 (10.95, 12.98)	**10.17 * (9.26, 11.16)**	**11.59 * (10.60, 12.65)**	0.976
Town	9.85 (8.95, 10.83)	**13.81 * (12.75, 14.93)**	**11.30 * (10.47, 12.18)**	11.58 (10.88, 12.32)	**9.76 * (9.16, 10.40)**	<0.001
Rural	7.55 (6.36, 8.95)	**12.71 * (11.32, 14.24)**	11.19 (10.08, 12.40)	12.23 (11.21, 13.33)	**10.22 * (9.35, 11.17)**	0.043
Socioeconomic status						
Less developed	7.28 (6.30, 8.41)	**9.71 * (8.39, 11.22)**	**7.04 * (6.12, 8.10)**	**8.50 * (7.47, 9.66)**	**7.26 * (6.37, 8.26)**	0.020
Moderately developed	9.78 (8.78, 10.88)	**14.26 * (13.23, 15.36)**	**11.65 * (10.86, 12.48)**	**12.89 * (12.20, 13.62)**	**10.71 * (10.14, 11.31)**	<0.001
Highly developed	8.98 (8.33, 9.69)	**10.28 * (9.67, 10.92)**	**11.56 * (10.92, 12.23)**	**9.97 * (9.49, 10.48)**	9.40 (8.94, 9.89)	0.030
Altitude						
<3km†	—	9.19 (7.77, 10.83)	10.51 (9.11, 12.11)	**14.32 * (12.98, 15.78)**	15.19 (13.81, 16.67)	<0.001
3-3.5km	11.19 (9.75, 12.82)	**14.97 * (13.55, 16.51)**	13.18 (11.93, 14.55)	12.60 (11.55, 13.72)	11.41 (10.46, 12.43)	0.002
3.5-4km	8.88 (8.24, 9.57)	**11.80 * (11.15, 12.48)**	11.82 (11.24, 12.43)	**9.60 * (9.15, 10.07)**	9.24 (8.83, 9.67)	<0.001
4-4.5km	7.05 (6.15, 8.06)	7.92 (7.05, 8.89)	**6.68 * (5.98, 7.45)**	**9.95 * (9.14, 10.81)**	**7.85 * (7.20, 8.56)**	0.006
≥4.5km	9.05 (7.65, 10.68)	**14.60 * (12.25, 17.32)**	**10.13 * (8.75, 11.69)**	10.41 (9.38, 11.53)	**7.04 * (6.30, 7.86)**	<0.001

Note. No data available for 2022. *CI*: Confidence interval. Bold and * indicate that there is a statistically significant difference in prevalence compared to the previous survey. † All participants in 2019 resided in areas above an altitude of 3 kilometers. Separate logistic regression models were fitted to assess subgroup standardized prevalence within strata of age, gender, school level, altitude, residence, and socioeconomic status, adjusting for the remaining covariates.

Table S9: Association between exposures of interest and DBM among children and adolescents in Xizang.

Variable	Undernutrition		Overnutrition	
	a*OR* (95% *CI*)	*P*	a*OR* (95% *CI*)	*P*
**Individual level**				
Fried food intake (Ref. Once a day or more)				
Less than once a day	0.989 (0.936, 1.044)	0.686	1.033 (0.967, 1.103)	0.334
SSB intake (Ref. Once a day or more)				
Less than once a day	1.003 (0.952, 1.057)	0.904	1.022 (0.961, 1.087)	0.481
Vegetable intake (Ref. Not Sufficient)				
Sufficient	0.948 (0.913, 0.985)	0.006	1.016 (0.971, 1.063)	0.491
Fruit intake (Ref. Not Sufficient)				
Sufficient	0.988 (0.953, 1.025)	0.530	1.029 (0.986, 1.074)	0.186
Breakfast habit (Ref. Not every day)				
Every day	1.003 (0.959, 1.049)	0.897	0.919 (0.873, 0.967)	0.001
MVPA (Ref. Not sufficient)				
Sufficient	0.937 (0.904, 0.972)	0.001	0.973 (0.934, 1.015)	0.205
**School level**				
Health education (Ref. Not launch)				
Launch	0.810 (0.715, 0.918)	0.001	0.739 (0.652, 0.838)	<0.001
Disease control (Ref. Not launch)				
Launch	0.904 (0.828, 0.987)	0.024	1.023 (0.928, 1.126)	0.652
Exercises between classes (Ref. Not sufficient)				
Sufficient	0.888 (0.817, 0.965)	0.005	1.019 (0.931, 1.114)	0.685
Annual sport meet (Ref. No)				
One time	1.032 (0.884, 1.203)	0.692	0.960 (0.823, 1.119)	0.600
≥ 2 times	0.955 (0.813, 1.123)	0.577	0.958 (0.814, 1.128)	0.608
School lunch menu (Ref. Not set)				
Set	0.922 (0.867, 0.980)	0.009	1.098 (1.024, 1.177)	0.008
Campus store (Ref. Set)				
Not set	1.051 (0.962, 1.148)	0.268	0.955 (0.868, 1.050)	0.342
**Social ecological level**				
Socioeconomic status (Ref. Less developed)				
Moderately developed	0.828 (0.725, 0.944)	0.005	1.138 (0.966, 1.339)	0.122
Highly developed	0.938 (0.818, 1.074)	0.354	1.269 (1.056, 1.525)	0.011
Altitude (Ref. <3km)				
3-3.5km	1.993 (1.375, 2.888)	<0.001	0.803 (0.601, 1.072)	0.136
3.5-4km	1.807 (1.259, 2.594)	0.001	0.616 (0.468, 0.813)	0.001
4-4.5km	2.491 (1.729, 3.588)	<0.001	0.478 (0.359, 0.637)	<0.001
≥4.5km	3.050 (2.057, 4.521)	<0.001	0.430 (0.315, 0.586)	<0.001
Ozone (continuous)	1.021 (1.014, 1.029)	<0.001	0.986 (0.979, 0.993)	<0.001
$PM_{2.5}$ (continuous)	0.973 (0.948, 0.999)	0.044	1.028 (1.001, 1.056)	0.045
Surface net solar radiation (continuous)	1.045 (1.014, 1.076)	0.004	1.013 (0.985, 1.041)	0.370
2m temperature (continuous)	0.956 (0.933, 0.979)	<0.001	1.037 (1.016, 1.059)	0.001
Total precipitation (continuous)	0.996 (0.994, 0.998)	<0.001	1.003 (1.001, 1.004)	<0.001

Note. *aOR*, adjusted odds ratio. *CI*, confidence interval. Ref, reference. SSB: sugar-sweetened beverages. MVPA: moderate-to-vigorous physical activity. SES: socioeconomic status. $PM_{2.5}$: particulate matter with an aerodynamic diameter ≤2.5 µm. *aOR* and their 95% *CI* were estimated by Multivariable Mixed-Effects Model analysis using normal nutrition as the reference category and including a random effect on the school and county. Model adjusted for survey year, age, gender, education level, residence, and family type. The analysis was conducted in N=128,642 individuals.

Table S10: Robustness analysis: Prevalence trends of undernutrition and overnutrition among children and adolescents in 10 consistently surveyed counties/districts from 2019 to 2024 (%).

Outcome	Prevalence (95% *CI*)					
	**Overall**	**2019**	**2020**	**2021**	**2023**	**2024**
Undernutrition	12.78 (12.55, 13.01)	16.72 (16.15, 17.31)	**14.08* (13.59, 14.60)**	13.67 (13.16, 14.21)	**10.49* (10.00, 11.01)**	**8.63* (8.21, 9.07)**
Stunting	10.90 (10.69, 11.12)	14.21 (13.68, 14.76)	**13.11* (12.63, 13.61)**	**12.23* (11.74, 12.74)**	**8.17* (7.73, 8.63)**	**6.31* (5.95, 6.69)**
Thinness	3.66 (3.53, 3.79)	4.95 (4.62, 5.29)	**2.91* (2.68, 3.17)**	**3.46* (3.19, 3.75)**	**3.96* (3.66, 4.30)**	**3.17* (2.91, 3.44)**
Overnutrition	11.83 (11.61, 12.06)	9.84 (9.39, 10.31)	**13.07* (12.59, 13.57)**	12.82 (12.31, 13.34)	12.75 (12.22, 13.31)	**10.65* (10.19, 11.12)**
Overweight	9.44 (9.24, 9.64)	7.76 (7.35, 8.18)	**10.39* (9.96, 10.84)**	10.20 (9.75, 10.67)	10.07 (9.59, 10.58)	**8.72* (8.30, 9.16)**
Obesity	2.40 (2.29, 2.50)	2.08 (1.87, 2.31)	**2.68* (2.45, 2.92)**	2.62 (2.38, 2.87)	2.68 (2.43, 2.96)	**1.93* (1.73, 2.15)**

Note. No data available for 2022. *CI*: Confidence interval. Bold and * indicate that there is a statistically significant difference in prevalence compared to the previous survey.

Table S11: Robustness analysis: Association between exposures of interest and DBM among children and adolescents in Xizang using complete case analysis.

Variable	Undernutrition		Overnutrition	
	a*OR* (95% *CI*)	*P*	a*OR* (95% *CI*)	*P*
**Individual level**				
Fried food intake (Ref. Once a day or more)				
Less than once a day	0.994 (0.939, 1.051)	0.826	1.047 (0.977, 1.121)	0.192
SSB intake (Ref. Once a day or more)				
Less than once a day	0.999 (0.946, 1.054)	0.962	1.020 (0.957, 1.087)	0.545
Vegetable intake (Ref. Not Sufficient)				
Sufficient	0.955 (0.918, 0.993)	0.021	1.022 (0.976, 1.071)	0.354
Fruit intake (Ref. Not Sufficient)				
Sufficient	0.986 (0.949, 1.023)	0.453	1.025 (0.980, 1.071)	0.281
Breakfast habit (Ref. Not every day)				
Every day	1.006 (0.961, 1.054)	0.792	0.913 (0.867, 0.963)	0.001
MVPA (Ref. Not sufficient)				
Sufficient	0.931 (0.897, 0.967)	<0.001	0.976 (0.934, 1.019)	0.264
**School level**				
Health education (Ref. Not launch)				
Launch	0.762 (0.669, 0.869)	<0.001	0.693 (0.604, 0.793)	<0.001
Disease control (Ref. Not launch)				
Launch	0.865 (0.790, 0.948)	0.002	1.004 (0.907, 1.111)	0.942
Exercises between classes (Ref. Not sufficient)				
Sufficient	0.866 (0.795, 0.944)	0.001	1.046 (0.952, 1.150)	0.346
Annual sport meet (Ref. No)				
One time	1.030 (0.877, 1.211)	0.717	0.918 (0.786, 1.074)	0.285
≥ 2 times	0.945 (0.798, 1.118)	0.508	0.924 (0.783, 1.089)	0.344
School lunch menu (Ref. Not set)				
Set	0.946 (0.887, 1.009)	0.091	1.087 (1.015, 1.165)	0.017
Campus store (Ref. Set)				
Not set	1.124 (1.015, 1.244)	0.025	0.860 (0.775, 0.955)	0.005
**Social ecological level**				
Socioeconomic status (Ref. Less developed)				
Moderately developed	0.793 (0.688, 0.914)	0.001	1.144 (0.964, 1.358)	0.123
Highly developed	0.738 (0.633, 0.859)	<0.001	1.468 (1.205, 1.788)	<0.001
Altitude (Ref. <3km)				
3-3.5km	1.988 (1.353, 2.922)	<0.001	0.805 (0.600, 1.080)	0.148
3.5-4km	1.738 (1.199, 2.521)	0.004	0.608 (0.459, 0.804)	<0.001
4-4.5km	2.412 (1.659, 3.509)	<0.001	0.481 (0.360, 0.643)	<0.001
≥4.5km	3.006 (2.000, 4.518)	<0.001	0.428 (0.313, 0.586)	<0.001
Ozone (continuous)	1.015 (1.007, 1.022)	<0.001	0.985 (0.977, 0.992)	<0.001
$PM_{2.5}$ (continuous)	1.009 (0.990, 1.029)	0.363	1.002 (0.980, 1.025)	0.843
Surface net solar radiation (continuous)	1.048 (1.026, 1.070)	<0.001	0.993 (0.973, 1.014)	0.503
2m temperature (continuous)	0.958 (0.893, 1.028)	0.235	1.036 (1.017, 1.056)	<0.001
Total precipitation (continuous)	1.000 (0.999, 1.001)	0.924	1.001 (1.000, 1.002)	0.280

Note. *aOR*, adjusted odds ratio. *CI*, confidence interval. Ref, reference. SSB: sugar-sweetened beverages. MVPA: moderate-to-vigorous physical activity. SES: socioeconomic status. $PM_{2.5}$: particulate matter with an aerodynamic diameter ≤2.5 μm. *aOR* and their 95% *CI* were estimated by Multivariable Mixed-Effects Model analysis using normal nutrition as the reference category and including a random effect on the school and county. Model adjusted for survey year, age, gender, education level, residence, and family type. The analysis was based on a subset of the population with no missing values for any covariates included in the model.
